# Intraoperative indocyanine green fluorescence imaging in breast surgery

**DOI:** 10.1007/s00404-020-05582-7

**Published:** 2020-05-23

**Authors:** Friedrich Kühn, Jens-Uwe Blohmer, Maria Margarete Karsten

**Affiliations:** Department of Gynecology, Charité – Universitätsmedizin Berlin, Charitéplatz 1, 10117 Berlin, Germany

**Keywords:** Breast cancer, Breast surgery, Reconstructive surgery, Fluorescence angiography, Indocyanine green

## Abstract

**Background:**

Since postoperative complications after reconstructive breast surgery are often related to drastic increases of patient suffering and treatment costs, several devices were developed in order to avoid them. In this respect, the intraoperative fluorescence angiography with indocyanine green (ICG) provides promising results by detecting ischemic skin intraoperatively.

**Methods:**

Women who underwent reconstructive breast surgery at the breast center at Charité between April and December 2017 were included in the analysis. General patient characteristics, medical history, type of surgery, as well as postoperative parameters, complications and patient reported outcomes were compared between patients operated using ICG fluorescence angiography and conventionally operated patients.

**Results:**

Among 68 patients with breast reconstruction 36 (52.9%) were operated with the ICG angiography device and 32 (47.1%) without. No significant differences regarding patient demographics, medical history, and surgical procedure were found. Wound healing disorders occurred in 11.1% of the ICG group and in 9.4% of the control group. About 11% of both groups developed major complications which required revision surgery. Complication rates and patient reported outcome did not differ significantly. Across both groups, only the risk factor resection weight (≥ 500 g) was significantly associated with wound healing disorders (RR = 6.80; 95%CI 1.93–23.81; *p* = 0.022).

**Conclusion:**

The purchase of a device for intraoperative ICG angiography might not be reasonable for every breast center. Further research in a larger cohort and prospective manner should be done to determine if the addition of ICG to breast reconstructive surgery in the German setting really leads to improved patient care.

## Introduction

Postoperative complications after reconstructive breast surgery are still a common problem in the care of breast cancer patients [1]. Since complications such as wound healing disorders and necrosis can lead to additional surgery and implant loss resulting in hospitalization as well as a drastic increase in treatment costs, certain devices have been developed in order to reduce them. One method that provides promising results regarding the decrease of complication risks is the indocyanine green (ICG) intraoperative imaging (SPY Elite Fluorescence Imaging System, NOVADAQ Technologies Inc., Canada) [2]. In addition to subjective clinical judgement it enables the surgeon to detect areas with reduced perfusion in mastectomy skin flaps or autologous tissue transplants to objectively predict necrosis [3]. Therefore, it has the potential to improve the patient’s outcome by excising endangered tissue or adjusting the implant type as well as the surgical procedure [4–6]. There is data suggesting that treatment costs could be reduced by using the SPY system [7].

Due to the growing economic pressure on hospitals, cost reduction is becoming increasingly important. However, larger investments in surgical equipment and their cost–benefit-relation need to be carefully considered.

The aim of this study was to investigate whether our clinical impression is consistent with the results presented by other research groups and if the purchase of the device is reasonable considering its efficacy in an institution like ours.

## Methods

All women who underwent immediate or secondary reconstructive breast surgery from April to December 2017 in the Department of Gynecology at Charité – Universitätsmedizin Berlin were included. Their data was retrospectively collected. During this period the clinic was equipped with the earlier mentioned fluorescence angiography device which was used for a randomly selected part of the patient group (SPY group). The decision on the use of the device was made by the operating surgeons. Intraoperatively, patients in the SPY group received 0.1–0.3 mg/kg body weight indocyanine green (Verdye® 5 mg/ml) as intravenous bolus injection. The injection was given at the end of surgery to evaluate the perfusion of the operated area. Tissue with questionable perfusion in the ICG angiography was either removed or conservatively treated. When deciding if tissue has to be removed or not, surgeons relied on their subjective clinical judgement as no cutoff for the resection of ischemic tissue in ICG imaging was defined. Non reconstructive contralateral surgeries in bilateral operated patients were also included in the analysis because we assumed that this may increase the patient’s risk for complications similar to bilateral breast reconstructions [1].

The data from the clinic’s internal patient files (SAP, SAP SE, Germany) was collected with Excel (Office Excel 2016, Microsoft, USA) and then statistically analyzed using SPSS Statistics (SPSS Statistics Version 25, IBM Corporation, USA). The recently implemented patient reported outcome database (HRTBT Medical Solutions, Germany), a web-based system, was used to capture the patient reported outcome (PRO) [8].

Next to general patient characteristics like age, BMI, comorbidities, nicotine exposure and medical history regarding prior breast surgery, prior breast radiation and chemotherapy, clinical features like the type of surgery (nipple sparing mastectomy, skin sparing mastectomy, other – including follow-up resection, scar correction, implant exchange, secondary implant insertion, reduction mammoplasty) and the type of reconstruction (implant based, use of mesh, acellular dermal matrix or tissue expander, autologous tissue transfer) were noted. In case of implant based reconstruction the implant position, shape and size were recorded. The postoperative parameters analyzed were histologic tumor and nodal stage, wound healing disorders (defined as dehiscence ≥ 5 mm, infection (i. e., detection of bacteria), necrosis and total or partial loss of nipple areola complex, NAC), revision surgery (e. g. debridement, implant removal or exchange, haematoma evacuation) non-elective further operations, duration of follow-up (i. e. time between surgery and last appointment in the breast center) and the patient reported outcome. This was based on the QLQ-BR23 questionnaire developed by the European Organization for Research and Treatment of Cancer (EORTC).

In the calculation of breast specific parameters women who underwent bilateral surgery were only counted as half item for each breast which ensures that no information gets lost and that no patient is counted twice in any category. This was to not distort the overall analysis. Some patient numbers are given as decimals (e.g. 0.5) for this reason. In case of continuous variables, Shapiro–Wilk test was used to assess if they were normally distributed. Univariate comparisons between patients treated with the SPY System and conventionally operated patients were made using chi-square test or Fisher’s exact test for categorial variables, two-sample t-test for normally distributed continuous variables and Mann–Whitney-*U* test for not normally distributed continuous variables. The significance level was set to alpha = 0.05. No adjustments for multiple comparisons were done.

## Results

### Patients, demographics and surgery

68 patients matched the criteria. 36 (52.9%) of them underwent surgery with the SPY system and 32 (47.1%) were operated conventionally. As shown in Table 1, no significant differences between both groups regarding their age distribution, other general data and risk factors such as high body mass index, diabetes mellitus or nicotine abuse could be detected. The average age of women in the SPY group was 43.4 (standard deviation, SD: 10.7) and 46.5 (SD: 10.7) years in the control group.

**Table 1:** General patient data, medical history and surgery

Characteristics	SPY (*n* = 36)	Control (*n* = 32)	*p*-value
Age (years)			0.245^+^
Mean (SD)	43.4 (10.7)	46.5 (10.7)	
			0.808*
< 45	16 (44.4%)	16 (50.0%)	
≥ 45	20 (55.6%)	16 (50.0%)	
BMI (kg/m^2^)			0.280^$^
Median (IQR)	23.4 (21.3–26.1)	22.1 (20.8–26.5)	
			0.585*
< 25	20 (64.5%)	21 (72.4%)	
≥ 25	11 (35.5%)	8 (27.6%)	
*Missing*	*5*	*3*	
Pregnancies			0.197^§^
None	8 (22.2%)	8 (25.8%)	
1	12 (33.3%)	11 (35.5%)	
2	7 (19.4%)	10 (32.3%)	
≥ 3	9 (25.0%)	2 (6.5%)	
*Missing*	–	*1*	
Genetic predisposition for breast cancer			0.460^§^
None known	22 (61.1%)	17 (53.1%)	
BRCA-1/-2	13 (36.1%)	15 (46.9%)	
CHEK2	1 (2.8%)	0 (0.0%)	
Comorbidities			
Diabetes mellitus	0 (0.0%)	1 (3.1%)	0.478*
*Missing*	*1*	–	
Arterial hypertension	5 (14.3%)	2 (6.3%)	0.431*
*Missing*	*1*	-	
Smoker	4 (11.1%)	5 (16.1%)	0.723*
*Missing*	–	*1*	
Former smoker	8 (22.2%)	3 (9.7%)	0.202*
*Missing*	–	*1*	
Prior surgery^a^			0.808*
N (%)	13 (36.1%)	12.5 (39.1%)	
Type of prior surgery^a^			0.180^§^
BCS	6.5 (50.0%)	8.5 (68.0%)	
Mastectomy	5 (38.5%)	1 (8.0%)	
Other	1.5 (11.5%)	3 (24.0%)	
Local cancer therapy^a^			
Surgery	9.5 (73.1%)	9.5 (76.0%)	1.0*
Adjuvant radiation	6 (46.2%)	3 (24.0%)	0.370*
Surgery indication			0.610^§^
Carcinoma	23 (63.9%)	24 (75.0%)	
Prevention	10 (27.8%)	6 (18.8%)	
Other	3 (8.3%)	2 (6.3%)	
Side of surgery			1.0*
Unilateral	22 (61.1%)	20 (62.5%)	
Bilateral	14 (38.9%)	12 (37.5%)	
Type of surgery^a^			0.275^§^
NSM	18.5 (51.4%)	19 (59.4%)	
SSM	10 (27.8%)	4 (12.5%)	
SSM with NAC reconstruction	2 (5.6%)	5 (15.6%)	
Other	5.5 (15.3%)	4 (12.5%)	
Neoadjuvant cancer therapy			0.244*
N (%)	23 (63.9%)	24 (75.0%)	
Chemotherapy	8 (34.8%)	13 (54.2%)	
None	15 (65.2%)	11 (45.8%)	
Duration of surgery (h)			0.124^+^
Mean (SD)	2:43 (0:48)	2:24 (0:52)	
Resection weight (g)^a^			0.153^$^
Median (IQR)	260 (149.3–381.8)	254.5 (173–449.3)	
			0.692*
< 500 g	33.5 (93.1%)	25.5 (86.4%)	
≥ 500 g	2.5 (6.9%)	4 (13.6%)	
*Missing*	–	*2.5*	
Implant insertion^a^			0.197*
N (%)	28.5 (79.2%)	29.5 (92.2%)	
Implant shape^a^			0.023*
Round	0 (0.0%)	5.5 (19.3%)	
Anatomic	27.5 (100%)	23 (80.7%)	
*Missing*	*1*	*1*	
Implant position^a^			0.070*
Epimuscular	11 (38.6%)	18.5 (62.7%)	
Submuscular	17.5 (61.4%)	11 (37.3%)	
Implant volume (ml)^a^			0.398^$^
Median (IQR)	331.3 (257.5–420)	308.8 (185–448.1)	
Expander insertion^a^			0.434*
N (%)	5 (13.9%)	2 (6.3%)	
Expander shape^a^			–
Round	0 (0.0%)	0 (0.0%)	
Anatomic	5 (100%)	2 (100%)	
Expander position^a^			1.0*
Epimuscular	2 (40.0%)	1 (50.0%)	
Submuscular	3 (60.0%)	1 (50.0%)	
Expander volume (ml)^a^			0.833^$^
Median (IQR)	50 (50–225)	155 (-)	
Autologous reconstruction (TRAM)^a^	2 (5.6%)	0 (0.0%)	0.494*
Mesh insertion^a^	11 (30.6%)	6 (18.8%)	0.401*
ADM insertion^a^	4 (11.1%)	1 (3.1%)	0.360*
Lymph node intervention^a^			0.537^§^
N (%)	18.5 (51.7%)	16 (50.0%)	
SNB	15.5 (83.8%)	13 (81.3%)	
AS	2 (10.8%)	3 (18.8%)	
AD	1 (5.4%)	0 (0.0%)	
Subset with reduced perfusion in ICG fluorescence angiography^a^			
N (%)	11.5 (31.9%)	–	
Resection of tissue with reduced perfusion^a^			
Yes	4 (34.8%)	–	
No	7.5 (65.2%)	–	

Numbers in italics are missing values

*BCS* breast conserving surgery, *NSM * nipple sparing mastectomy, *SSM *skin-sparing mastectomy, *NAC *nipple areola complex, *SNB *sentinel lymph node biopsy, *AS *axillary sampling, *AD *axillary dissection, *ICG *indocyanine green, *ADM *acellular dermal matrix, *SD *standard deviation, *IQR *interquartile range

^a^In the calculation of breast specific parameters women who underwent bilateral surgery were only counted as half item for each breast which ensures that no information gets lost and that no patient is counted twice in any category

Test on normal distribution: Shapiro–Wilk test

^+^t-test for independent samples

^$^Mann–Whitney-*U* test

*Fisher’s exact test

^§^Chi-square-test

With one exception Table 1 also shows no significant differences between the two groups concerning operative features. The most frequent type of mastectomy was nipple sparing mastectomy (SPY: 51.4%; control: 59.4%) and the majority of patients (SPY: 79.2%; control: 92.2%) underwent an implant based reconstructive procedure. There was a significant difference between the groups concerning the implant shape. All patients with implant based reconstruction in the ICG group received anatomic shaped implants whereas 19.3% of the patients in the control group received round implants (Fisher’s exact test: *p* = 0.023). With regard to the implant position, inverse conditions were found: there were more patients with submuscular inserted implants in the SPY group (61.4%) compared to 62.7% epimuscular insertions in the control group. However, this difference was not statistically significant (Fisher’s exact test: *p* = 0.070). Reconstruction through autologous tissue transfer (TRAM) was performed in only two patients. Both of them received intraoperative ICG imaging.

**Fig. 1:**
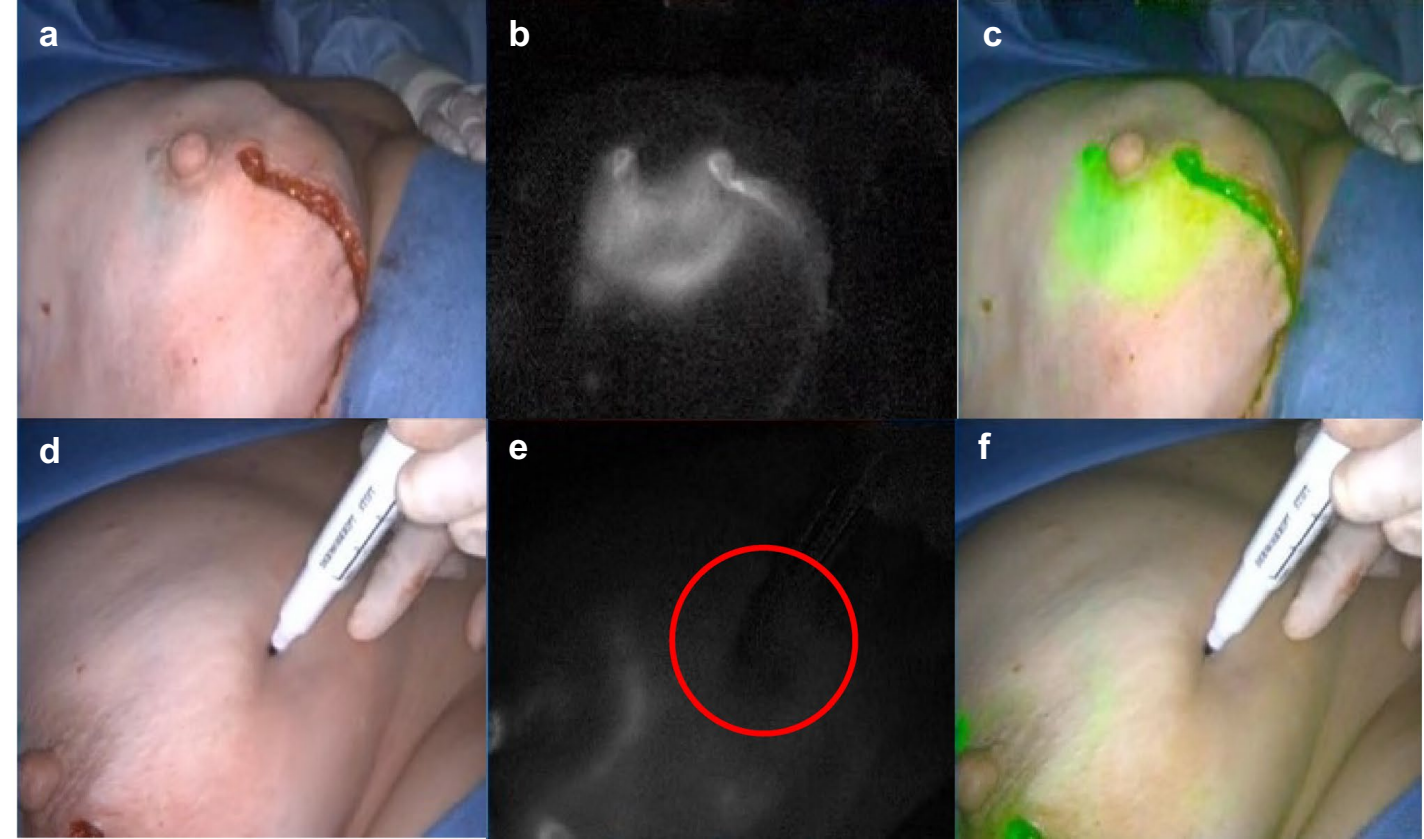
Intraoperative ICG imaging (**b**, **e**) shows reduced perfusion (circled dark region in picture **e**) after implant based reconstruction following nipple-sparing mastectomy in comparison to native intraoperative images (**a**, **d**). The SPY software enabled an overlay (**c**, **f**) of ICG imaging and native pictures. The upper (**a**, **b**, **c**) and lower (**d**, **e**, **f**) row of pictures show different perspectives on the reconstructed breast

In 11.5 patients of the SPY group (31.9%) a reduced perfusion was detected intraoperatively (Fig. 1). This led to a resection of the potentially endangered tissue in 34.8% (4 cases). The remaining 65.2% did not receive any further intervention and were treated conservatively.

### Postoperative course and follow-up

Table 2 demonstrates important postoperative parameters: 20 patients (55.6%) of the SPY group and 14 patients (43.8%) of the control group were registered in the PRO database. The length of the follow-up period and the number of registrations in the PRO database did not differ significantly between both groups. The distribution of tumor stage and nodal status of the cancer patients within the groups were significantly different. This information did not lead to a change of the surgical procedure or affect our primary endpoint. It is listed for completeness only.

**Table 2:** Postoperative parameters

Characteristics	SPY (*n* = 36)	Control (*n* = 32)	*p*-value
Histology			
N (%)	23 (63.9%)	24 (75.0%)	
Tumor stage			0.040^§^
cis	4 (17.4%)	2 (8.3%)	
T0	2 (8.7%)	10 (41.7%)	
T1	9 (39.1%)	10 (41.7%)	
T2	6 (26.1%)	2 (8.3%)	
T3	2 (8.7%)	0 (0.0%)	
Nodal status			0.048^§^
N0	16 (69.6%)	20 (83.3%)	
N1	7 (30.4%)	1 (4.2%)	
N2	0 (0.0%)	1 (4.2%)	
NX	0 (0.0%)	2 (8.3%)	
Resection			0.497^§^
R0	19 (82.6%)	22 (91.7%)	
R1	3 (13.0%)	2 (8.3%)	
RX	1 (4.3%)	0 (0.0%)	
Follow-up (d)^b^			0.820^$^
Median (IQR)	84 (29–181)	96.5 (26–157)	
Patient reported outcome			0.466*
Included	20 (55.6%)	14 (43.8%)	
Not included	16 (44.4%)	18 (56.3%)	

*cis* carcinoma in situ, *IQR *interquartile range

^b^The follow-up time is the time between surgery and last appointment in the breast center

Test on normal distribution: Shapiro–Wilk test

^$^Mann–Whitney-*U* test

*Fisher’s exact test

^§^Chi-square-test

Complication rates are shown in Table 3. Relating thereto no significant differences were found. The most common complication was seroma in 21 (58.3%) patients of the SPY group and 15 patients (46.9%) in the control group. 3 patients (9.4%) who were conventionally operated and 4 (11.1%) patients operated using ICG angiography developed a wound healing disorder. Of the 4 patients whose critically perfused tissue identified by ICG imaging was removed only 0.5 (one breast of a bilateral operated patient, 12.5%) showed wound healing issues despite excision. Ischemia was detected around the 3-point in this case but a dehiscence with accompanying infection could not be prevented. Major complications requiring revision surgery occurred in 4 cases of each group (SPY: 11.1%; control: 12.5%).

**Table 3:** Complications

Characteristics	SPY (*n* = 36)	control (*n* = 32)	*p*-value
Wound healing disorder^a^			1.0*
N (%)	4 (11.1%)	3 (9.4%)	
Dehiscence^c^	2 (50.0%)	2 (66.7%)	1.0*
Necrosis	3 (75.0%)	3 (100%)	1.0*
Accompanying infection^d^	2 (50.0%)	1.5 (50%)	1.0*
Loss of NAC			0.350^§^
partial	1 (25.0%)	0 (0.0%)	
total	1 (25.0%)	0 (0.0%)	
Wound healing disorder of area with reduced perfusion in ICG fluorescence angiography^a^			
N (%)	11.5 (31.9%)	–	
Yes	2.5 (21.7%)	–	
No	9.0 (78.3%)	–	
Wound healing disorder after resection of tissue with reduced perfusion in ICG angiography^a^			
N (%)	4 (11.1%)	–	
Yes	0.5 (12.5%)	–	
No	3.5 (87.5%)	–	
Other complications^a^			
Seroma	21 (58.3%)	15 (46.9%)	0.466*
Haematoma	1.5 (4.2%)	1.5 (4.7%)	1.0*
Need of revision surgery^a^	4 (11.1%)	4 (12.5%)^e^	1.0
Time to first revision (d)			0.133^+^
Mean (SD)	14.8 (7.9)	38 (29.7)	
Further surgery^a^			0.761*
N (%)	6 (16.7%)	6.5 (20.3%)	
Non-elective	4 (66.7%)	4.5 (69.2%)	1.0*
Unplanned antibiotics	7 (19.4%)	6 (18.8%)	1.0*

*NAC* nipple areola complex, *ICG* indocyanine green, *SD* standard deviation. ICG was applied as intravenous bolus (0.1–0.3 mg/kg body weight). Perfusion time and washout time could not be determined retrospectively. Subjective clinical judgement led either to resection or preservation of tissue indicated as ischemic by ICG imaging

^a^In the calculation of breast specific parameters women who underwent bilateral surgery were only counted as half item for each breast which ensures that no information gets lost and that no patient is counted twice in any category

^c^Dehiscence ≥ 5 mm

^d^Detection of bacteria

^e^One revision caused by haematoma

test on normal distribution: Shapiro–Wilk test

^+^t-test for independent samples

*Fisher’s exact test

^§^Chi-square-test

Concerning the patient reported outcome Table 4 shows the survey results of breast specific questions in EORTC’s QLQ-BR23 questionnaire. From the SPY group 16 out of 20 (80.0%) women and from the control group 8 out of 14 (57.1%) women registered in the PRO database took part in the postoperative survey. The median time between surgery and survey was 12.9 (IQR 6.2–25.5) weeks in the ICG group and 9.4 (IQR 6.5–21.3) weeks in the control group. This difference was not found to be significant (Mann–Whitney-Test: *p* = 0.881). The mean breast symptom score of the ICG group was 26.5 (SD: 22.5) and 30.3 (SD: 24.8) in the control group. Although there were obvious numerical differences, none of the scores resulted in a statistically significant difference between the two groups.

**Table 4:** Patient reported outcome EORTC’s QLQ-BR23

Characteristics	SPY (*n* = 36)	Control (*n* = 32)	*p*-value
Subset registered in PRO database			
N (%)	20 (55.6%)	14 (43.8%)	
Participated	16 (80.0%)	8 (57.1%)	
*Missing*	*4*	*6*	
Time since surgery (weeks)			0.881^$^
Median (IQR)	12.9 (6.2–25.5)	9.4 (6.5–21.3)	
BR23 scores			
Body Image			0.447^+^
Mean (SD)	62.4 (25.7)	70.9 (24.0)	
Breast symptoms^f^			0.713^+^
Mean (SD)	26.5 (22.5)	30.3 (24.8)	
Arm symptoms^f^			0.620^+^
Mean (SD)	34.6 (18.7)	30.4 (21.2)	

Numbers in italics are missing values

*PRO* patient reported outcome, *SD* standard deviation, *IQR* interquartile range

^f^Higher scores indicate a lower quality of life

test on normal distribution: Shapiro–Wilk test

^+^t-test for independent samples

^$^Mann–Whitney-U test

Table 5 indicates that there is no significant association between a critical perfusion detected by ICG imaging and the development of wound healing complications (Fisher’s exact test: *p* = 0.304). In the SPY group 1.5 (37.5%) patients showed wound healing disorders which were not predicted by the device. In the remaining 2.5 (62.5%) patients they appeared in the area previously indicated. 5.5 of the 7.5 (73.3%) cases where skin was not debrided, despite reduced perfusion in ICG imaging, did not develop healing complications. In the univariate evaluation of the common risk factors a significant association between the development of a wound healing disorder and a resection weight of 500 g or more was shown for the entire cohort (RR = 6.80; 95%CI 1.93–23.81; *p* = 0.022). Since the used implant shape differed significantly between the groups, tests on a possible correlation of implant shape and wound healing disorder were conducted. No association was found in this regard (Fisher’s exact test: *p* = 1.0).

**Table 5:** Possible influencing factors

Characteristics	No wound healing disorder (*n* = 61)	Wound healing disorder (*n* = 7)	*p*-value	Relative risk	95% CI
Smoker			0.327*	1.76	0.34–9.26
No	52.5 (87.5%)	5.5 (78.6%)			
Yes	7.5 (12.5%)	1.5 (21.4%)			
*Missing*	*1*	–			
Former smoker			1.0*	0.85	0.11–6.36
No	50 (83.3%)	6 (85.7%)			
Yes	10 (16.7%)	1 (14.3%)			
*Missing*	*1*	–			
Previous radiation^a^			0.582*	not applicable**	
No	52 (85.2%)	7 (100%)			
Yes	9 (14.8%)	0 (0.0%)			
Overweight (BMI ≥ 25 kg/m^2^)			0.418*	2.16	0.54–8.54
No	37.5 (70.8%)	3.5 (50.0%)			
Yes	15.5 (29.2%)	3.5 (50.0%)			
*Missing*	*8*	–			
Age (≥ 45 years)			1.0*	1.13	0.29–4.90
No	29 (49.2%)	3 (42.9%)			
Yes	32 (52.5%)	4 (57.1%)			
High resection weight (≥ 500 g)^a^			0.022*	6.80	1.93–23.81
No	55 (94.0%)	4 (57.1%)			
Yes	3.5 (6.0%)	3 (42.9%)			
*Missing*	*2.5*	–			
Previous surgery^a^			1.0*	0.93	0.21–4.02
No	38 (62.3%)	4.5 (64.3%)			
Yes	23 (37.7%)	2.5 (35.7%)			
Bilateral surgery			0.700*	0.65	0.14–3.09
No	37 (60.7%)	5 (71.4%)			
Yes	24 (39.3%)	2 (28.6%)			
Subset with implant insertion^a^					
N (%)	52.5 (86.1%)	5.5 (78.6%)			
Implant shape anatomic^a^			1.0*	not applicable**	
No (round)	5.5 (10.9%)	0 (0.0%)			
Yes	45 (89.1%)	5.5 (100%)			
*Missing*	*2*	–			
Subset with ICG fluorescence					
N (%)	32 (52.5%)	4 (57.1%)			
Reduced perfusion in ICG fluorescence^a^			0.304*	3.54	0.53–23.81
No	23.0 (71.9%)	1.5 (37.5%)			
Yes	9.0 (28.1%)	2.5 (62.5%)			
Resection of tissue with reduced perfusion^a^					
N (%)	9 (28.1%)	2.5 (62.5%)	1.0*	0.43	0.03–8.13
No	5.5 (61.1%)	2.0 (80.0%)			
Yes	3.5 (38.9%)	0.5 (20.0%)			

Numbers in italics are missing values

*ICG* indocyanine green, *CI* confidence interval. ICG was applied as intravenous bolus (0.1–0.3 mg/kg body weight). Perfusion time and washout time could not be determined retrospectively

^a^In the calculation of breast specific parameters women who underwent bilateral surgery were only counted as half item for each breast which ensures that no information gets lost and that no patient is counted twice in any category

**The sample size is too small for an appropriate approximation by "the rule of three"

*Fisher’s exact test

## Discussion

This retrospective study could not detect any significant differences between the two surgical approaches in regard to complication rates and patient reported outcomes of a representative patient cohort in a highly specialized breast center of a German university clinic. In both groups wound healing disorders occurred in about 10% of the cases. The incidence rate of revision surgery was similar (11.1% and 12.5%). No significant associations were found, even though the majority of wound healing disorders (62.5%) in the SPY group were predicted by the device. 87.5% of the cases where ischemic tissue was removed after ICG imaging had uncomplicated recovery without further wound healing issues. Nevertheless, it is not certain whether they were actually prevented by the intervention or whether they would not have appeared in the first place: 73.3% of the patients where the alleged ischemic skin was preserved did not show any healing complications. The fairly high seroma rate in our patient cohort in both arms is due to the fact that we routinely do ultrasound examinations so even small seromas not requiring intervention are noted.

Our observation, thus, differs from other published results [5, 7, 9, 10]. According to Jones, postmastectomy skin necrosis occurred in 4.3% of the reconstructions with tissue expanders [3]. He assumes that a reduction of the necrosis rate to 0% could have been achieved by a consequent resection of the tissue which was found to be less perfused since all necroses appeared in the area identified as ischemic by ICG imaging [3]. Komorowska-Timek et al. could show that by excising the endangered tissue detected through ICG angiography overall complication rates decreased from 15.1% to 4% [2]. In this respect, Newman et al. describe a 95% correlation between ICG imaging and clinical outcome with a specificity of 91% and sensitivity of 100% [11]. Phillips et al. also report a correct prediction in 90% of their cases [4]. Mastectomy flap necrosis occurred less frequently in a retrospective evaluation by Harless et al. after the implementation of ICG angiography with an incidence of 0.9% instead of 6.7%. Their overall complication rate was significantly reduced as well after integrating ICG imaging into their clinic [5]. Duggal et al. were able to reduce necrosis rates by 10.4% percent and revision operations also had to be performed in 5.9% instead of 14.1% only. In addition, they present an analysis revealing a significant cost reduction for breast reconstruction through fluorescence angiography [7].

However, the comparison with the previously listed studies is only partially valid in our case. Each of them was conducted in the United States where the surgical approach is different from ours. In our clinic, mastectomy and reconstruction are both performed by the same surgeon (gynecologist). In the U. S., usually a breast surgeon and a plastic surgeon are involved [2–5, 7, 11]. In addition, skin-sparing mastectomy and tissue expander based or autologous reconstructions were more common in the American setting [2–5, 7, 11]. The majority of our patients underwent nipple-sparing mastectomy (NSM) and reconstructions were mainly conducted using permanent implants. The local differences aside and given that ICG imaging led to resection in only 4 cases, the necrosis rate (SPY: 8.3%, Control: 9.4% and 8.8% of the total cohort) in our observation was slightly lower than mentioned in the literature [12]. A possible explanation could be that the breast surgeon in our clinic is also responsible for the reconstruction. A reconstructive surgeon performing mastectomy might treat the remaining tissue more carefully than a surgeon whose primary concern is only oncologic safety. We could not verify an association in this regard, but the increased risk of skin flap necrosis after NSM when the surgery is done by two different surgical teams might explain the smaller benefit we could achieve by using ICG in our setting [12].

Apart from the earlier cited publications there are studies that take a critical look at the use of the device. Of the 62 breast reconstructions conducted by Munabi et al., 8 cases (13%) developed a necrosis that could only be predicted with 88% sensitivity and 83% specificity by the SPY system since several events were overpredicted in patients with nicotine exposure or epinephrine injection [13]. Therefore, the authors doubt the accuracy of ICG imaging and recommend particular caution for smokers and the use of epinephrine containing tumescent solution to avoid overcorrection (i.e., unnecessary excision of actually intact tissue) [13]. Mattison et al. also describe a high sensitivity, but a low specificity of only 68% with a low positive predictive value (34.8%). This is due to a high rate of false positives. In many cases the region assessed to be less perfused by ICG imaging was significantly larger than the area determined by the subjective clinical assessment of the surgeon. The authors fear that this could lead to an inappropriate resection of healthy tissue, which would negatively influence the duration of tissue expansion, wound healing and treatment costs [14]. In contrast to our evaluation, there was a significantly lower rate of severe necrosis requiring revision surgery in the setting of Diep et al. with 4.9% in the ICG group and 18.9% in the control group (*p* = 0.02). However, they were not able to reach a significant reduction of their overall necrosis rates [10]. Regarding treatment costs, Kanuri et al. described that the expenses for prosthesis-based reconstructions of high risk patients including smokers, obese patients and patients with a high mastectomy weight could be reduced by using the SPY system. Yet, no financial benefit could be found for patients without those risk factors [15]. On the basis of these publications, it must be assumed that the device is only suitable for high-risk patients and should be used with particular caution to protect healthy tissue, to ensure the aesthetic result and to not endanger the patient’s safety [16].

As a retrospective evaluation, the study has limitations. In order to gain a broad clinical impression of the device's potential applications, we opted for rather loose inclusion and exclusion criteria. This led to a relatively heterogeneous patient cohort concerning mastectomy, reconstructive procedures and medical history. The group allocation (i.e., the selection of the surgical method) was not randomized and operations were performed by six different surgeons. Also, perfusion and washout time of ICG, as well as standardized criteria for skin evaluation/resection could not be determined retrospectively. As shown in a retrospective evaluation by Diep et al. the implementation of ICG angiography improved outcomes of post-mastectomy reconstructions only over time [17]. They could not yet describe a concrete learning curve but stated that a certain case volume per surgeon was necessary for an improvement [17]. All of the involved surgeons in our clinic were not familiar with the device and there are no consistent guidelines yet, concerning the threshold between viable and ischemic tissue in ICG imaging. This is why they preferably relied on their clinical judgement regarding the removal of ischemic skin. Due to the small number of cases and postoperative events, multivariate analyses could not be conducted. Therefore, only univariate analyses were done.

Since the implementation of the web based patient reported outcome measurement coincided with the observation period, the inclusion rate was not a hundred percent [8]. The postoperative breast symptom and arm symptom score seem to be rather high, but a decrease is to be expected in the further course. There is no official cutoff but in relation to the reference values of Langendijk et al. who published scores after a median time since surgery of 5 years, our scores shortly after surgery seem appropriate [18].

## Conclusions

The purchase of a device for intraoperative ICG angiography for our specific setting needs to undergo further examination in a larger cohort since no difference could be demonstrated in overall outcome, complication rates and patient reported outcome. Further (prospective, randomized) studies and proper guidelines in regard to excision of tissue for all surgeons involved are necessary to adequately verify the usefulness of ICG imaging.
